# Healthcare Professionals’ Experiences with Vocational Rehabilitation for People with Inflammatory Arthritis

**DOI:** 10.1007/s10926-025-10297-0

**Published:** 2025-05-13

**Authors:** Anne-Birgitte Madsen, Jette Primdahl, Jeanette Reffstrup Christensen, Ann Bremander, Christina Merete Tvede Madsen

**Affiliations:** 1 Occupational Therapy Education & Department for Applied Health Science, Esbjerg, Denmark; 2 Danish Centre for Expertise in Rheumatology, Danish Hospital for Rheumatic Diseases, University Hospital of Southern Denmark, Engelshøjgade 9 A, 6400 Sønderborg, Denmark; 3 Department of Regional Health Research, University of Southern Denmark, Odense, Denmark; 4 Hospital Sønderjylland, University Hospital of Southern Denmark, Aabenraa, Denmark; 5 Research Unit of General Practice, Department of Public Health, University of Southern Denmark, Odense, Denmark; 6 DRIVEN—Danish Centre for Motivational and Behavior Science, Department of Sports Science and Clinical Biomechanics, University of Southern Denmark, Odense, Denmark; 7 Research Unit of General Practice, Aarhus, Denmark; 8 Section of Rheumatology, Department of Clinical Sciences Lund, Lund University, Lund, Sweden

**Keywords:** Axial spondyloarthritis, Complex intervention, Psoriatic arthritis, Rheumatoid arthritis, Work rehabilitation, WORK-ON

## Abstract

**Purpose:**

To explore healthcare professionals' (HPs’) experiences of work-related challenges among people with inflammatory arthritis (IA).

**Methods:**

A qualitative, interview study using a hermeneutic approach was planned. HPs with different professional backgrounds working with people with rheumatic diseases were recruited. An interview guide was developed according to relevant literature. The analysis followed Graneheim and Lundman’s qualitative content analysis.

**Results:**

Twenty-one HPs representing two municipalities, three hospitals, a university college and one patient organisation participated in individual semi-structured interviews. The analysis derived three themes: (1) *Work identity and living with IA*. The disease causes emotional and economic effects regarding fulfilling roles in everyday life, including work; (2) *Opportunities and challenges when supporting patients.* Promoting and inhibiting factors that affect retention in the labour market include organisational factors at work, opportunities for involving the patient’s relatives and working interprofessionally and cross-sectorally; and (3) *Cooperation with employers*. People with IA use different strategies and opportunities for compensatory schemes to maintain work. Cooperation with employers is an important part of vocational rehabilitation.

**Conclusion:**

HPs experience that people with IA find it difficult to manage their everyday life, including work. HPs want to support people with IA to maintain their jobs but find it difficult if the patient has not informed the employer about the disease. This study clarifies the need for vocational rehabilitation to support people with IA to stay in work, from time of diagnosis through hospitalisation, municipal rehabilitation and job clarification.

## Introduction

In Denmark, approximately 50,000 are diagnosed with rheumatoid arthritis and 30,000 with psoriatic arthritis or axial spondyloarthritis [1]. In this paper, these diagnoses are referred to as chronic inflammatory arthritis (IA). Despite the constant development of new pharmacological treatments, IA may still lead to pain, joint stiffness, decreased mobility and fatigue, causing physical limitations as well as psychosocial challenges in everyday life including work [2–4].

Work contributes to a sense of normality, good health and quality of life [4, 5]. People with IA experience work disabilities to a greater extent than the general population and may experience challenges balancing energy in everyday life, including work [2, 6–8]. They are challenged by work demands such as working full time, repetitive work tasks and physically demanding tasks, and find it difficult to participate in work at the same level as employees without IA. Furthermore, fatigue is experienced as a major challenge for concentration, creativity and planning [8, 9]. These challenges may lead to low work ability, long-term sick leave and low quality of life, and up to 38% of people with IA lose their jobs within the first few years after being diagnosed [10–15]. Because of reduced work ability, a diagnosis of IA may lead to economic consequences for the individual as well as for society [6, 16]. Therefore, it is important to offer effective vocational rehabilitation (VR) to people with IA with the aim of increasing their work ability and preventing sick leave and job loss.

A systematic review of six studies examining job loss prevention interventions among people with IA concluded that VR may improve work ability and reduce sick leave and job loss [14]. The results were inconclusive, however, given the diversity of the interventions and variety of outcome measures. Moreover, the interventions were sparsely described, making it difficult to replicate them. In addition, VR varies across countries because of differences in legal and social security systems. Consequently, the review concluded that it is necessary that VR is tailored to the specific context [14].

VR can be considered a complex intervention because it involves various healthcare professionals (HPs), from both secondary and primary social and healthcare sectors [17]. The British Medical Research Council’s framework for the development and test of complex interventions emphasises the importance of involving relevant stakeholders in the development process [17]. To inform the development of a new context-specific VR, WORK-ON, in Denmark, we have explored perceived challenges at work and the need for professional support among people with IA [8]. Likewise, it is important to investigate HPs’ perspectives on their need for support to maintain work among people with IA to inform the development of a new context-specific VR. Thus, the aim of this study was to explore HPs’ experiences of work-related challenges among people with IA.

## Methods

### Design

We planned a qualitative, explorative interview study using a hermeneutic approach to seek in-depth understanding of HPs’ perspectives of work-related challenges concerning the need for VR among people with IA [18, 19].

### Participants and Recruitment

Managers at three hospitals, two municipalities, one university college and a patient organisation, all located in Denmark, were contacted by email with an inquiry to identify HPs working with people with rheumatic diseases, who would be willing to participate in an interview. Possible participants were contacted by phone or email with information about the study. If they did not respond to the email, they were contacted by phone. If they were interested in participating, they signed a written consent and a time and place (physical, phone or online) for an interview were agreed upon.

### Data Collection

According to the previous systematic review [14], interviews with people with IA [8], input from patient research partners and a study exploring the experiences of HPs delivering VR [20], an interview guide was developed encompassing the following topics: Challenges at work among people with IA, Patients’ ability and opportunity to balance work with other activities in everyday life, Social aspects influencing the patients’ work ability and Vocational rehabilitation (Table 1). We performed semi-structured interviews with all included participants [19]. These interviews were conducted from June 2020 to October 2020 and were held physically, by phone or online, depending on the participants’ preferences.

**Table 1:** All topics and some examples of questions in the interview guide

Topic	Examples of questions
Challenges at work among people with IA	Which challenges do you experience that people with IA face in their work? How do you assess what the patient’s challenges are?
Patients’ ability and opportunity to balance work with other activities in everyday life	Which opportunities do you have to support the citizens/patients to manage their energy throughout the day?
Social aspects influencing the patients’ work ability	Do you have the opportunity to involve the patient’s workplace (employer, colleagues)?
Vocational rehabilitation	What are your best experiences with vocational rehabilitation?

*IA* Inflammatory Arthritis

### Context and Legislation

In Denmark, citizens with impaired work ability who are at risk of job loss can be supported by a municipal job centre. Employers are only informed about the employees’ health condition if the employee discloses it. There are various opportunities for support and compensatory schemes, offers in the municipal job centres and possibilities to apply for a flexi-job. A flexi-job means that the municipality pays a subsidy to the employer if the person can only work less than half time (in Denmark, full time is 37 hours/week). Another opportunity is §56, which is a support scheme that secures the employer reimbursement from the municipality for missed workdays. To keep a §56 agreement, the person needs to use it at least 10 days per year. Employees also have the option to be on partial sick leave from work if they are not ready to return to their normal working hours. These schemes, and maybe a job training programme, can help determine how many hours the person is able to work.

### Data Analysis

The interviews were conducted by Christina Merete Tvede Madsen (CMTM) and were recorded and transcribed verbatim by CMTM and Anne-Birgitte Madsen (ABM). The transcriptions were checked for accuracy by ABM. The analysis of the transcribed interviews followed Graneheim and Lundman’s qualitative four-step content analysis to derive both a manifest and a latent level [21].

The analysis moved from a concrete description to an abstract interpretation in accordance with the hermeneutic approach. First, all interviews were read to gain an overview of the content. Second, each interview transcript was read again to extract meaning units relevant to the study aim. Third, these meaning units were condensed, coded and organised into subcategories and categories. Fourth, the content within these categories was interpreted, progressing from a manifest to a latent level, resulting in the formulation of overarching themes supported by explanatory text. To enhance the trustworthiness of the findings, a stringent and transparent data analysis was applied. Throughout the analysis, the whole author group engaged in discussions to ensure consensus. The analysis was iterative, involving movement between different stages and considerations of both parts and the whole. NVivo version 12 software was used for data organisation and analysis, contributing to a systematic and transparent approach [18, 21, 22].

## Results

Twenty-one HPs (19 women and two men) were interviewed. The participants’ characteristics are summarised in Table 2. Six interviews were conducted physically, 12 by telephone and one online. The interviews lasted between 43 and 70 min.

**Table 2:** Characteristics of the of HPs (*n* = 21)

Characteristic	Number
Gender Female (%)	19 (90)
Work experience in years; median, (range)	20 (2–38)
Workplace Hospital (in-patient rehabilitation) Municipality* University college Patient organisation	10 5 1 5
Profession Physician (rheumatology and occupational medicine) Social worker Psychologist Physiotherapist Occupational therapist Nurse Researcher	3 8 1 1 4 3 1

*Including job centre and healthcare centre

**Fig. 1:**
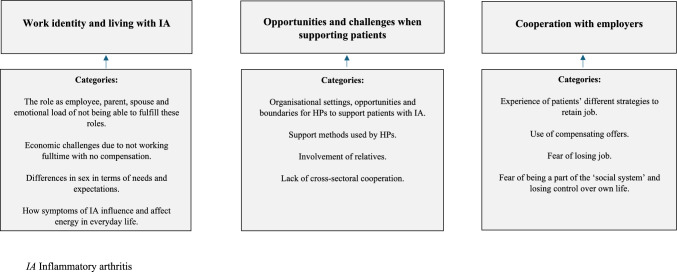
Themes and categories. *IA* Inflammatory arthritis

The analysis derived three overall themes: (1) *Work identity and living with IA*, (2) *Opportunities and challenges when supporting patients* and (3) *Cooperation with employers.* In Fig. 1, the categories and themes are illustrated graphically. These themes are described in more detail and illustrated by selected interview quotations in the following sections. Finally, suggestions for support are described.

### Work Identity and Living with IA

The HPs reported that people with IA are challenged in their everyday lives and that, although work plays an important role for them, it may take energy from other roles in everyday life, such as being a parent or a partner. The HPs considered that fatigue is the main challenge for patients with IA to maintain their job, although they really want to stay at work. In addition, the HPs highlighted that people with IA often express concern about letting down their colleagues and employer if they are not able to perform their work in the same way they used to. HPs reported that they had the impression that people with IA have less energy left for family, children, parents or partner and that it becomes a struggle to engage in social activities. HPs noticed that this lead to people with IA feeling guilt towards the partner and family, shame for not being able to fulfil their roles and a negative self-image and self-perception. Problems to fulfil a role as spouse can also lead to concerns about their partner’s patience towards commitment and, thus, the relationship is strained.

The HPs considered that having a job and being part of a workplace are associated with improved quality of life and contributes to financial security for people with IA. People with IA do not want to be seen as different or burdensome at the workplace because they are afraid of being considered ‘weak’. This can mean that they refrain from asking for help.

Further, the HPs noted that people with IA often mentioned concerns about economic challenges. For instance, they stated that although a part-time job would fit them given their challenges, this cannot be afforded.

*We need to offer an alternative to them, so that they (people with IA) do not have to go from a full-time employment (37 hours per week) to less than 25 hours per week, because if you must be economic self-sufficient, not many people would be able to do that, if they don´t have a partner who earns a good salary.* (Social worker, hospital).

The HPs also noted differences in how women and men cope with their disease to maintain a job. They observed that men tend to suppress pain, overlook discomfort and avoid complaining about their situation, whereas women often discuss the challenges they experience at work as well as at home. The HPs found that the role of provider can inhibit men with IA from talking about their problems and may also affect the support they receive.

*Generally, women are better at expressing their challenges […] might be due to classic gender roles […] as we expect of the men to provide […] I think it lies deep within the men, especially men over 50, that complaining, particularly if they are in an environment where it is difficult for them to say that they have difficulties buttoning the shirt […] I think it is more socially acceptable for women to speak out.* (Physiotherapist, hospital).

### Opportunities and Challenges when Supporting Patients

In general, the HPs identified it as a challenge that some people with IA choose not to disclose their IA to their employer or colleagues because of uncertainty whether the employer will react negatively, including the risk of being fired or looked at differently by coworkers. The HPs considered this led to the patient not receiving the necessary support, such as workplace accommodations, assistive devices or agreements on breaks or flexible working hours.

*I ask them if they have any assistive devices, if there is a problem, and how the manager’s attitude is towards these things, and whether the colleagues know about it, it is substantial information to have […] if they haven’t told anybody […] the workplace misses the opportunity to create good conditions.* (Occupational therapist, hospital).

HPs found it challenging not being able to provide the needed support to people with IA in situations where they choose not to disclose their IA. These situations often resulted in prolonged overload experienced by the person with IA, followed by the need for full-time sick leave. HPs highlighted it as beneficial if the patient’s relatives were involved in meetings with HPs because they enable understanding and provide complementary insights into how the disease manifests in everyday life and when accommodations in everyday life are necessary. Not all HPs had formalised involvement of relatives, but all did inform the person with IA about the possibility of such involvement. Nonetheless, although HPs recognised that ensuring support from relatives helped provide people with IA with the best conditions, they rarely met relatives in their daily practice. HPs expressed that the involvement of relatives needs further considerations, given that relatives may support people with IA, positively influencing their attachment to the labour market.

*There are a lot of patients who receive very good help and support for practical matters if they choose to stay in the workforce and prioritise that. […] we talk a lot about the importance of daring to open to your relatives and ask for help.* (Nurse, hospital).

Lack of cross-sectoral collaboration can be an obstacle for coherence in the professional initiatives taken to support patients with IA. This collaboration may involve the hospital and various municipal departments, such as the job centre and rehabilitation centre. Some HPs had the impression that cross-sectoral and interprofessional collaboration had decreased over time, and that they no longer had the time to participate in interdisciplinary meetings across sectors. For instance, HPs from the hospital often attempted to facilitate cross-sectoral communication post-discharge because timely coordination prior to discharge was not always possible. This results in a communication gap between the time of discharge and the initiation of support in the municipality.

*I often think that it is a challenge to get in touch with social workers in the municipality in the short time we have before they are discharged. Because it might not be the case that the social worker calls back in the time range […] then I´ll do it the week after and then afterwards I can call the patient.* (Social worker, hospital).

The HPs noted that lack of collaboration between sectors poses a barrier to patients’ rehabilitation process, given that initiatives started at the hospital are not always continued after discharge. People with IA may also have difficulties following up on goals on their own.

*Yes, I get so happy when someone is admitted down there [hospital] [...] but when they get home again, everything comes to a halt. People really benefit from it and feel better physically, and mentally for that matter, but when they come home, it just stops. It’s such a shame.* (Social worker, municipality).

Occupational therapists and physiotherapists at hospitals develop rehabilitation plans that facilitate cross-sectoral contact with HPs in the municipality. However, lack of knowledge about the municipalities’ resources prevents some HPs from developing rehabilitation plans.

*I think that we […] could be better at making rehabilitation plans […] but I actually think it’s because we are uninformed, and we don’t know what the municipality can do or when we can actually take action.* (Occupational therapist, hospital).

The HPs also considered that interprofessional collaboration can be challenging because of a lack of time and opportunities to meet. Brief in-person meetings would mean that rehabilitation plans could be transferred more effectively.

*I think our patients might also feel like they are almost given a pile of papers, or that it’s all just stored in their heads, but that it doesn’t really connect, or that we assume it makes sense to them—but I don’t always think it does. […] The best experience, I believe, is when we have looked at it interdisciplinary, developed something together and provided the patient with something concrete that they can also work with.* (Occupational therapist, hospital).

### Cooperation with Employers

HPs reported that people with IA often did not use the compensatory scheme §56, which may lead to withdrawal of the scheme. HPs observed that people with IA found it difficult to make use of their §56 agreement because they may consider it a sign of vulnerability and expect that its use would affect their colleagues and employer negatively. These concerns often involve fear of job loss and permanent exclusion from the labour market. HPs thought that cooperation with employers may lead to other compensatory agreements that could be useful. For instance, agreements between employers, affected employees and HPs from the municipality job centre may enable adjusting working time to make it possible to start the day later or allow employees to rest during the workday to reduce pain and fatigue, including having employers arrange a place to rest in the workplace. In general, HPs noticed that a thorough agreement regarding work pace and work tasks is very important. In addition, when HPs attend meetings with both the employee and the employer, they can provide information about legislation and opportunities for the workplace. Overall, if people with IA are open to finding solutions at the workplace, the HPs thought that they could be implemented to support them to stay at work. Employers should also inform all relevant colleagues about new agreements that may increase successful implementation. Some HPs found that when people with IA do not want to inform their employer about the disease it could be because of a lack of acceptance of the disease. This also poses a barrier for positive cooperation with the employer.

*Some of those who have physically demanding jobs are very worried if their hands won’t cooperate or if they are in a lot of pain and if they have a static job where they do the same thing all the time. It’s hard on their arthritis. So, they are concerned about whether they’ll be able to manage their job and fear that when they take a sick day, it might have consequences. It’s really a concern for their working life and their future, which is also uncertain.* (Nurse, hospital).

HPs reported that people with IA often have a strong fear of becoming a part of the social system and losing control over their work life. Some HPs explained how some people with IA had mentioned that they were afraid of engaging in a long-term job clarification process. Further, some people with IA had disclosed that, when they had tried to use the social system, navigating it was challenging and resource-intensive for them and that they henceforth tried to avoid it.

*This week, I think I’ve spoken with four people who have expressed this fear of getting in touch with the job centre and going through a long job clarification process, and I encounter that from time to time. So, it’s not just about navigating the system; they simply don’t dare. Some of them have been in contact with the job centre before and say, ‘I just can’t go through that again.’* (Social worker, patient organisation).

HPs reported that it was difficult for patients with IA to accept and make use of the option for partial sick leave.

*But a partial sick leave can help them get some peace in mind […] they don’t want that, though. They hold on to the belief that they’ll manage to get back up again if they are on sick leave, and before they start telling anyone, it might have already been five days […] but they are very reluctant to show that this is something they probably can’t just sleep off, medicate or get surgery for. They keep hoping...* (Social worker, municipality).

HPs found it challenging that people with IA tend to work more than they are actually able to manage. Thus, HPs encourage them to use the compensatory schemes. The HPs also noted that the best sustainable solution is early intervention.

## Discussion

This study has explored HPs’ experiences of work-related challenges among people with IA. We found that the HPs experienced that people with IA struggle to maintain their work identity and fulfil different roles in everyday life, such as being a worker, spouse and parent. The difficulty in juggling different roles in everyday life, including work, is supported by other studies exploring perspectives from patients with IA [23, 24]. HPs themselves experienced a range of challenges when supporting patients with IA to maintain work, including interprofessional and cross-sectoral cooperation and the involvement of relatives. The HPs in the current study emphasised that involving relatives in clinical practice was beneficial, as the relatives facilitate understanding and offer complementary insights into how the disease manifests in everyday life and when adjustments are needed. This is in line with other studies highlighting that relatives provide valuable input, but relatives may also affect the patient with IA both positively and negatively [25, 26]. An increased awareness of including relatives is therefore necessary.

Cooperation with employers was considered essential because it allows implementation of adjustments at the workplace, but often people with IA do not disclose their disease to their employer and coworkers. These findings resonate with previous research exploring perceived challenges at work among people with IA [8, 24]. For instance, people with IA may not disclose having the disease because they do not want to be stigmatised, seen as ‘the sick one’ or as a burden [27–29]. People with IA may also lack the required knowledge to guide disclosure or to understand the implications early disclosure may have in supporting their work ability and being able to access support from the employer [8, 24]. Other studies exploring employers’ perspectives have shown that employers actually want to be involved in employees’ rehabilitation processes to support the person in the best possible way, which would require that the employer is aware of employees having IA [30–33]. That is, to be able to implement adaptation at work, employers need to be informed about the work-related challenges their employees may face. The HPs in the present study suggested that materials with information about IA in terms of causes, symptoms, prognosis and possible effects on functioning could help people with IA disclose information to their employers.

HPs in this study highlighted challenges in cooperation between hospitals and municipalities due to a lack of time. This is in line with another study from Denmark that investigated HPs’ experiences and approaches to care coordination across sectors when older people are acutely hospitalised [34]. This study also found several challenges in cooperation between sectors associated with a lack of time, resources and task coordination [34]. Another Danish study explored how health and social care professionals across different departments, levels and sectors work to achieve coherent rehabilitation pathways for people with IA [35]. The study found that HPs are dependent on cooperation with other HPs across departments and sectors, and that the institutional and organisational framework can hinder rather than promote the HPs’ intentions to create coherent rehabilitation plans [35]. The study pointed towards the importance of a person-centred approach rather than a system approach focused on the organisational and legal constraints [35]. Similar findings were reported in another study concerned with neuromuscular diseases that investigated community-based HPs’ reflections on and behaviours concerning collaboration with a tertiary rehabilitation hospital [36]. These studies may indicate general barriers for cooperation between sectors in the Danish healthcare system of which HPs should be aware. To accommodate this challenge, HPs need to work with a person-centred approach, which means, as suggested by Feddersen et al. [35], meeting patients with an individual, respectful and holistic approach. In addition, future collaboration in rehabilitation models should be interdisciplinary and team based. The findings from Handberg et al. highlight the importance of strengthening the structure of collaborative team dynamics [36]. Doing so may lead to well-planned, coordinated and effective rehabilitation, while also supporting future cross-sectoral collaboration [36].

Job loss has the potential of causing a crisis in the individual’s self-understanding. This is because work is not only a source of economic self-sufficiency, but also gives meaning and structure to everyday life and social relations [4]. To handle this crisis, Giddens [37] suggests that the individual seeks new ways to find meaning and structure and builds a new identity through new job opportunities, education or voluntary work. This is in line with our findings given HPs reported that people with IA fear job loss. This reinforces the need for people with IA to receive support to help them maintain their job for as long as possible through adjustments at work and compensatory schemes, but also to reorient themselves towards other opportunities. This includes alternative job opportunities, educational opportunities or voluntary work, allowing a focus on values and identity through which a new identity can be developed.

## Implications

It is important to disseminate knowledge for employers and people with IA about compensatory schemes, legislative offers and the potential for people with IA to remain at work. If needed, patients with IA should receive support to disclose their disease to their employer and thus get access to legislative support schemes. For employees with IA, this may help them to maintain work as an important part of their identity and mental and social aspects of health and may help reduce sick leave and job loss. HPs also need more knowledge about support opportunities in the municipalities.

Further, based on the HPs experiences they suggested that VR should support people with IA through the process of informing their employer and colleagues about their situation and disease. They suggested written materials about opportunities for support in terms of legislation, compensatory schemes and cooperation with the workplace. They stressed that cooperation between the employer and employee is important for making agreements in the workplace and that the employer has a key role in informing their colleagues. They also suggested the inclusion of materials with information about IA in terms of causes, symptoms, prognosis and possible effects on functioning and that relatives should be involved in VR.

## Strengths and Limitations

It is a strength of this study that participants encompassed HPs from different types of hospitals, various municipalities and municipal departments and the Danish Rheumatism Association. It was also a strength that telephone interviews were used when necessary, allowing access to more HPs. At the same time, telephone interviews may be considered a limitation because they limit data to verbal communication. A limitation can also be found in that it was only possible to recruit one psychologist with rheumatology experience. Finally, this study focused solely on HPs’ perspectives, but it would be beneficial to explore employers’ perspectives in the national context to complement the findings.

## Conclusion

HPs have noted that people with IA find it difficult to manage their everyday life, including work, and that this affects their identity. HPs want to support people with IA maintain their jobs but find this difficult if the patient has not disclosed their disease to their employer. HPs find cooperation with employers crucial for implementing new agreements and compensatory schemes as needed. Interprofessional and cross-sectoral collaboration and communication may also be a challenge because of a lack of time and of opportunities to meet and communicate in a timely fashion. HPs see potential in the inclusion of relatives to support in the VR process. This study clarifies the need for a person-centred approach and for VR to support people with IA to stay in work, encompassing diagnosis through to hospitalisation, municipal rehabilitation and job clarification. VR requires cross-sectoral and interprofessional collaboration, communication and coordination.

## Data Availability

The data analysed are not available because of the need to preserve anonymity.
